# Differences in training and practice in tunnelled haemodialysis catheter removal: a survey of nephrology trainees across United Kingdom

**DOI:** 10.1186/s12882-025-04034-1

**Published:** 2025-03-06

**Authors:** Ismet Boral, Shalabh Srivastava, Joanna McKinnell

**Affiliations:** 1 University Hospitals of Derby and Burton NHS Foundation Trust, Derby, UK; 2 South Tyneside and Sunderland NHS Foundation Trust, Sunderland, UK; 3 Newcastle University, Newcastle-upon-Tyne, UK

**Keywords:** Nephrology, Training, Survey, Haemodialysis, Catheter removal, Central venous access, UK

## Abstract

**Background:**

The tunnelled haemodialysis catheter (TDC) removal is a necessary skill for the nephrology trainee as this task is undertaken routinely in renal units. Little published data exists to establish current practice and there is no national guidance regarding TDC removal in United Kingdom (UK). Anecdotally, trainees suggest they do not have sufficient supervised training in TDC removal. We aimed to establish the differences in training and practice in TDC removal among nephrology trainees across UK.

**Method:**

We created an online survey with twenty questions for trainee and non-training nephrology registrars working in UK. The survey was distributed via regional renal training programme directors, UK Kidney Association, “Renal SpR Club” and online professional social networks including social media and instant messaging services.

**Results:**

We received 75 responses from all of 14 postgraduate training deaneries. 91% reported renal registrars remove TDCs in their units. 53% of the operators were taught by another registrar. Only 16% report awareness of written local trust guidance on TDC removal. 43% reported removing > 10 TDCs a year. Cut-down method is preferred over traction method for TDC removal. 63% remove TDCs in designated procedure areas, 52% obtain written consent and 65% wear full sterile personal protective equipment (PPE). 16% report removing TDCs alone with no assistant and 12% do not stop aspirin, antiplatelets or anticoagulants beforehand. 30% of operators reported experiencing a “stuck catheter” at some point in their careers.

**Conclusions:**

This survey highlights that TDC removal is a common procedure and predominantly performed by renal physicians in teaching hospitals. It is mostly undertaken by registrar level doctors often without formal training or written guidelines with varying techniques. 68% of participants want this procedure to be part of mandatory training in the renal post graduate training curriculum.

**Trial registration:**

Not applicable.

**Supplementary Information:**

The online version contains supplementary material available at 10.1186/s12882-025-04034-1.

## Background

The removal of tunnelled haemodialysis catheter (TDC) is a necessary basic skill for the nephrology trainee as this task is undertaken routinely in renal units after successful establishment of other permanent access or due to need for removal in catheter-related blood stream infections. Anecdotally, most trainees suggest that they do not have sufficient supervised training in this clinical procedure, yet this procedure forms a regular part of their clinical work.

We would like to establish the differences in training and practice in TDC removal among nephrology trainees across UK.

### Nephrology training

Currently nephrology speciality training in the UK is paired with general internal medicine training and it runs for 4 years. The training years are named from ST4 to ST7 for speciality trainee (ST) years. Speciality doctors are also known as registrars. The doctors who act in the registrar role but not in the Royal College of Physicians approved training pathway are designated non-training or trust grade registrars. The renal training curriculum (version 2022) only includes non-tunnelled dialysis catheter insertion as a mandatory procedure for training. Training in TDC removal is not a mandatory or desired skill in the current or previous renal curriculae. There is no definition on how to train and how to remain competent on this procedure.

### TDC removal procedures

There are two major different techniques of TDC removal described in literature. These are the cut down method (CDM) and traction method (TM). Both techniques are practiced by the authors. There are also reported modifications of either technique.

CDM is in summary a new incision made over the cuff, followed by blunt dissection through the new incision down to and around the cuff before clamping and cutting the catheter into two parts and removing them separately [1]. Palpation is used to locate the cuff and a sterile field is created around the cuff area by cleaning the skin with chlorhexidine or iodine. A sterile drape is applied. Local anaesthetic is inserted to the skin above the Dacron cuff, then a 2–3 cm incision to the skin is made over the cuff. The operator uses blunt dissection down to the cuff and around the cuff to liberate it from the surrounding tissue. The catheter is clamped to prevent air embolism and cut distal to the cuff. This allows the distal external portion to be removed through the exit site. Then the proximal intravascular component is pulled out and immediate pressure is applied to the vein entry point. In the modified CDM (MCDM), after the clamp is applied, the intravascular part is pulled out first to minimise the risk of catheter migration or retention. Once haemostasis is achieved and the incision site stitched the drape can be removed and finally the distal catheter portion is removed through the exit site [2].

Traction method is where the TDC is removed through the exit site in toto [3, 4]. Any sutures are removed. The exit site, surrounding skin and external portion of the catheter are cleaned with chlorhexidine or iodine solutions. Sterile drape is applied to achieve a sterile operating field. The local anaesthetic is applied to the tunnel all the way from the exit site to the Dacron cuff. The operator then blunt dissects with a variety of surgical instruments (e.g. hemostat clamp) through the exit site to reach the cuff, all the while applying traction on the external portion of the catheter with the other hand. The cuff is then liberated from the surrounding tissue with further blunt dissection and the whole catheter is pulled out with a carefully applied vigorous force in toto. In situations where the cuff is more than 2 cm away from the exit site, the use of transcatheter extractor was also described in a very small number of patients [5].

### Complications of the procedure

Porazko et al. 2020 [2] report 14% risk of any post procedure complication in 143 patients where CDM or MCDM was performed. The most frequent complications were prolonged bleeding followed by wound infection. There were two cases of catheter migration and a single case of air embolism reported in the group where the catheter was clamped and cut before the intravascular component was removed.

Fulop et al. 2017 [6] summarise all of their relevant previous work with a combined total of 265 TDC removals with TM. These studies were Fulop et al. 2013 [4] (55 patients), Fulop et al. 2015 [7] (138 patients), Dossabhoy et al. 2016 [8] (72 patients). They report two cases of prolonged bleeding across all three studies and nine cases of cuff retention in the study with 138 subjects. There was one stuck catheter adherent to superior vena cava in the series reported by Dossabhoy et al. They report not encountering any catheter fracture, catheter migration or air embolism across three studies. It is worth noting that they only removed double lumen catheters. Kohli et al. suggests some catheters e.g. Tesio are more likely to fracture with traction [3].

One of the most dreaded complications of TDC removal is the “stuck catheter” which is the adherence of the catheter to central veins or right atrium. In a retrospective review of a single-centre experience with more than two thousand TDC removals, this complications was reported to be 0.92% [9]. The stuck catheter may be extracted by a variety of techniques including surgical [10], endoluminal balloon dilatation [11, 12] or by advancing an introducer sheath over the catheter [13]. There are other reported ways of extracting a stuck catheter summarised by Forneris et al. [14].

There is a risk of catheter fracture and retention of catheter fragments. This is rare but serious complication as it can embolise in the vasculature [15]. There is also a risk of longer parts of the catheter migrating into the central venous system during removal [16]. 

Air embolism after catheter removal is a risk but most published reports involve non-tunnelled dialysis catheters. As discussed above Porazko et al. reported a single case in their series where the catheter was cut before the intravascular part was removed. Theoretically removing the catheter from the vessel before cutting it mitigates this risk. Fulop et al. 2017 suggests the tunnel collapses after removal of tunnelled catheters in traction method, preventing air entry.

### Guidance on TDC removal

Even though this procedure poses significant problems with rare but major complications, there are no specific easy to find national or international guidelines on TDC removal. The literature appears to be focusing on insertion, maintenance and management of dysfunction or infections. UK Kidney Association (UKKA) published a guideline in April 2023 on “vascular access for haemodialysis” [17]. This includes some suggestions on catheter insertion and care but none on removal. Kidney Disease Outcomes Quality Initiative (KDOQI) published their own vascular access guideline in April 2019 [18]. Again, this guideline includes recommendations on the insertion technique, timing of TDC removal but makes no suggestions on how to remove TDCs. Finally, American Society of Diagnostic and Interventional Nephrology (ASDIN) provides no freely available guidelines on TDC removal either.

## Methods

We designed an electronic survey with twenty questions to establish the landscape in training and practice in TDC removal across UK. This survey was specifically developed for this study and it is provided as supplementary file 1. This survey was designed by one nephrology trainee and two nephrology consultants. It was targeted at nephrology registrars, mainly aimed at trainees but non-training grade doctors were welcomed to participate. Survey questions were piloted locally in Royal Derby Hospital before wider dissemination. Seventeen questions were mandatory to complete the survey with three optional questions. Two questions were free-text answer only. The rest of the questions had multiple choice answers with some of them having a free-text final choice available to minimise restrictions. The questions covered participant training grade, their workplace location, their previous training, level of experience and their practice specifics in TDC removal procedure.

The study aim and the rationale were explained in the introduction of the survey and the participants were advised that they would be giving their consent to participate in this study by completing the survey. All participants gave their informed consent to participate in the study by completing the electronic survey. The electronic system does not allow submission of partially completed surveys. The participants are anonymous. No incentives or rewards for participation were offered. Ethical approval was not required according to Health Research Authority under paragraph 2.3.14 of UK policy document GAfREC (governance arrangements for research ethics committees) last updated and published on 20 July 2021.

We distributed this survey via different channels. We approached all regional renal training programme directors (TPD) twice via The Joint Royal Colleges of Physicians’ Training Board (JRCPTB) and asked them to forward the survey link to their registrars in the region. The survey was also advertised on the front page of UK Kidney Association (UKKA) website for one month. We contacted “Renal SpR club” which supports networking between renal trainees in the UK and Ireland. They shared the survey link on their social media channels. Finally, we reached out to personal contacts across the country and asked for the survey to be shared in relevant registrar groups on social media and instant messaging services. The survey was active and accepted responses for approximately 7 months from 1st January 2024 to 22nd July 2024.

The quantitative data was analysed with statistical functions on Microsoft Excel 2016 (Microsoft Corporation) and relevant graphs were drawn on the same software. Power calculations and significance calculations were not possible due to the nature of the data and the small sample size. Therefore, the results are mainly expressed in percentages. The free text answers were analysed by using the principles of qualitative data analysis.

## Results

### Training grade and region

75 nephrology registrars responded across 14 postgraduate training deaneries in UK. The distribution of registrars and their regions are in Table 1. The base hospital of the registrars was an optional question and 29 different answers were given.

**Table 1:** The distribution of nephrology registrars across regions and training levels. ST; specialist trainee

Regions	Not in training	ST4	ST5	ST6	ST7	Total
East Midlands	2	6		5	3	**16**
East of England				2		**2**
Kent, Surrey and Sussex					1	**1**
London		1	1	4		**6**
North East		1			4	**5**
North West			1			**1**
Northern Ireland	1			1		**2**
Scotland		2	2	1	1	**6**
South West		3		1	1	**5**
Thames Valley	1	1	1	1	1	**5**
Wales			2	5	3	**10**
Wessex			2		1	**3**
West Midlands	1		2	1		**4**
Yorkshire and Humber		2	3	2	2	**9**
Total	**5**	**16**	**14**	**23**	**17**	**75**

### Operators in the TDC removal procedure

**Fig. 1:**
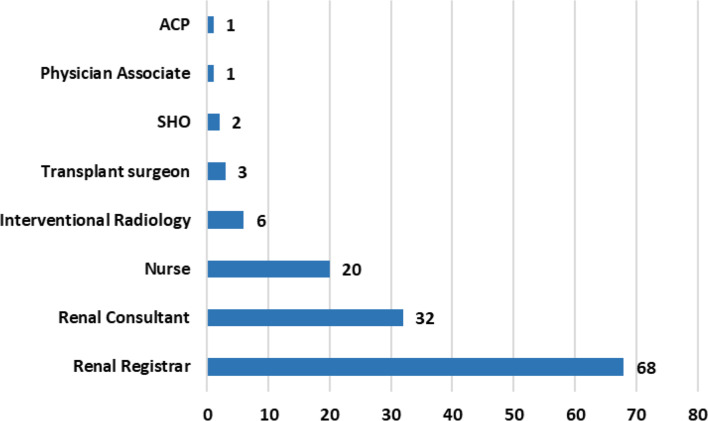
Who removes TDCs in your current renal unit? Select all that apply. ACP; Advanced Care Practitioner, SHO; Senior house officer (an older term used to describe doctors who are more junior than speciality registrar level)

68 participants (91%) report that renal registrars are involved in TDC removal in their respective units (Fig. 1).

### Training of registrars on this procedure

**Fig. 2:**
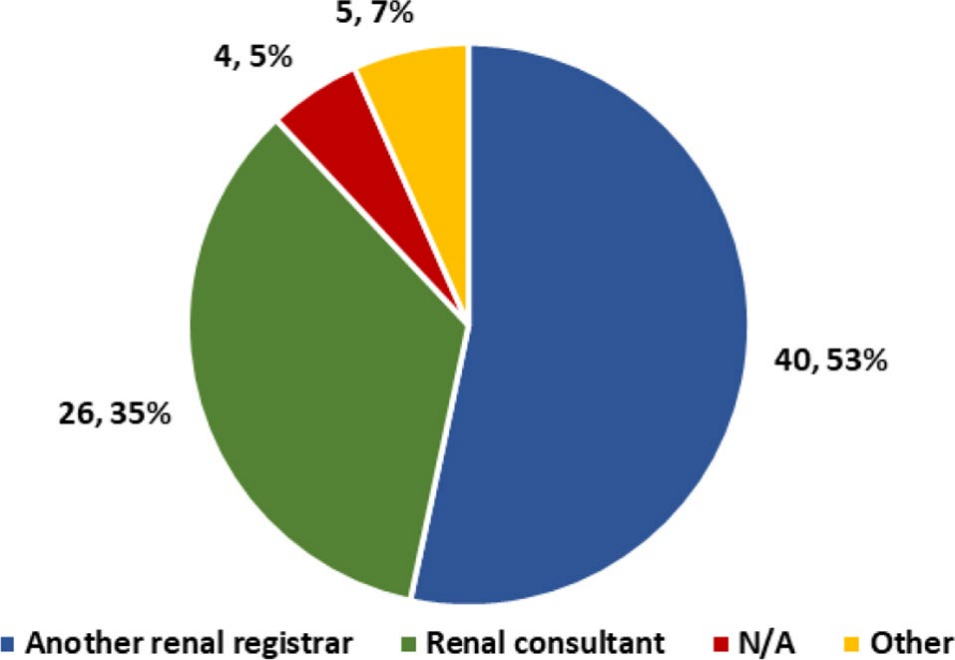
Who taught you how to remove TDCs?

53% learnt the procedure from another registrar and 35% from a renal consultant (Fig. 2).

### Awareness of local guidelines on TDC removal

Only 16% of operators report awareness of local trust guidelines on TDC removal. 36% report they don’t have guidelines and 48% suggest they are unsure as they never had to check.

### Experience levels

55 participants (73%) report they have removed more than 10 TDCs in total so far with 18 (24%) reporting > 50 previous procedures. 43% of all participants report that they remove > 10 TDC a year. 4 participants (5%) reported not performing this procedure yet.

### The brand of TDC

**Fig. 3:**
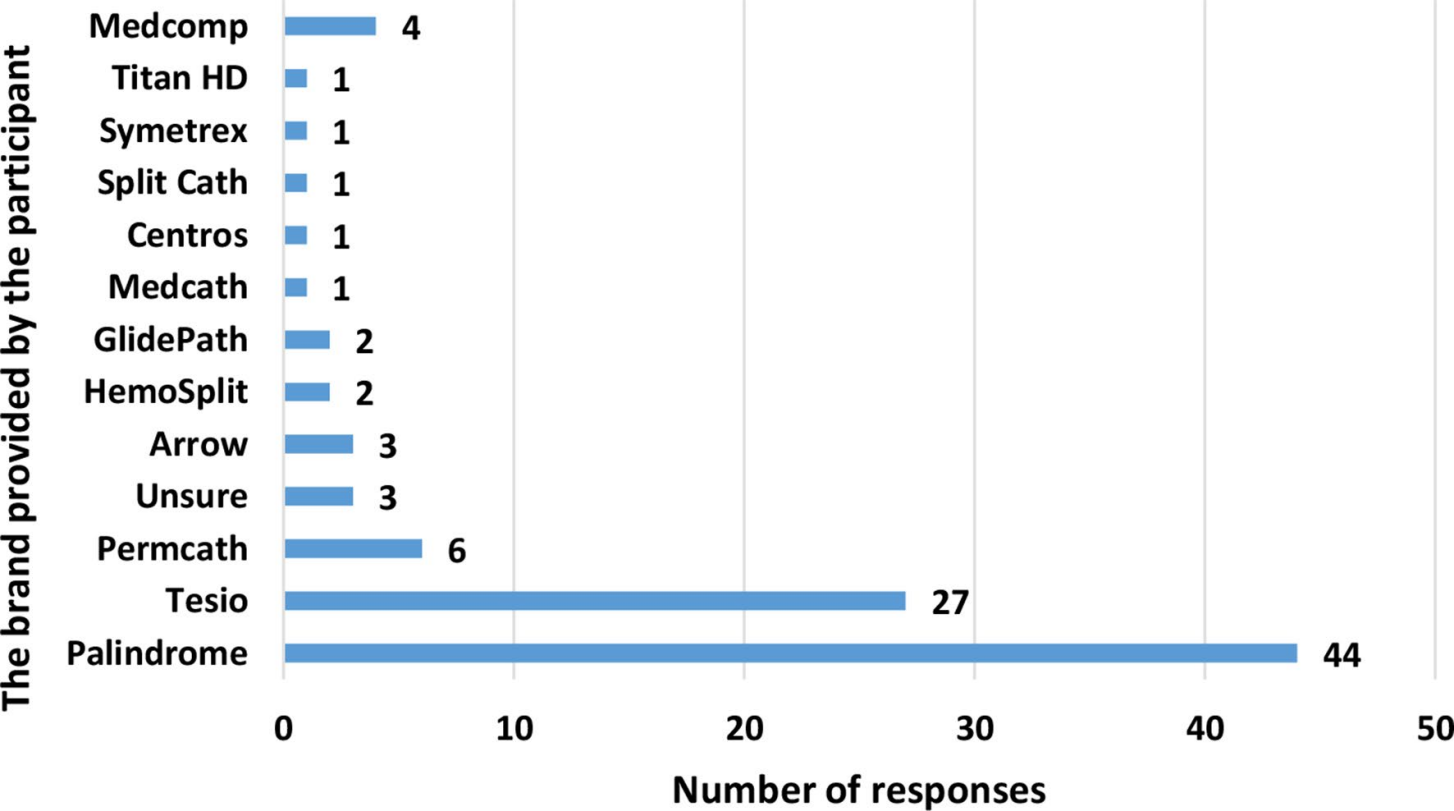
There may be differences in the nature of the lines used, necessitating different ways of removal. What type of tunnelled dialysis catheters do you use?

This was an optional question with multiple answers allowed. When asked about what brand of TDC they removed, 37 participants reported one single brand, 24 participants gave more than one answers and 3 responded “unsure”. The results are shown in Fig. 3. It should be noted that 4 participants responded with “Medcomp” in their free-text answer however Medcomp is a company whose chronic haemodialysis catheter products include Titan HD, Tesio, Split Cath, Symetrex and more.

### Cut down vs. Traction methods

37% report that they mostly perform CDM, 19% report they prefer TM, 39% report they can perform either technique but choose depending on the need with no strong preference. The last 5% are not trained to do either procedure yet. As a sub analysis, half of registrars who prefer the traction method work in East Midlands with the other half being split across Northern Ireland, Scotland, Yorkshire and Humber.

### Designated area

63% report they operate only in a designated procedure area and 12% only by the bedside. The rest report both. Designated procedure areas appear to be available in most cases.

### Management of anticoagulants and antiplatelets pre-procedure

**Fig. 4:**
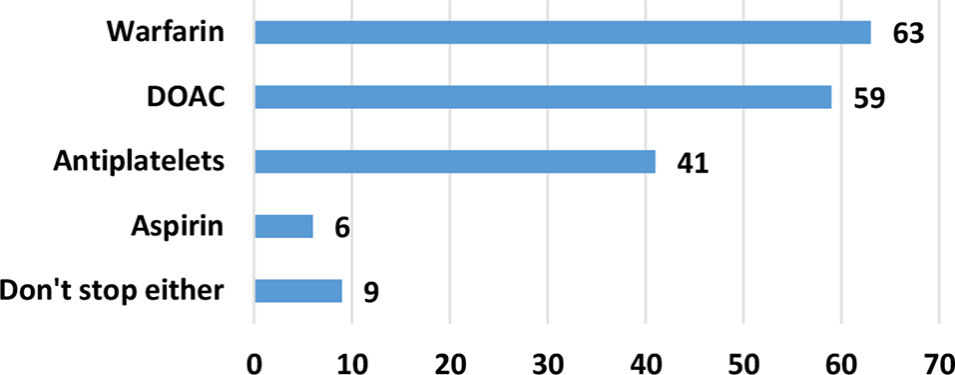
Do you ask for these medications to be stopped in an elective TDC removal? We accept that when stopped they are stopped for at least 5 days before the procedure

Warfarin and direct oral anticoagulants (DOACs) are stopped by most operators before the procedure, 89% and 83% respectively. 58% stop antiplatelets including clopidogrel, prasugrel and ticagrelor before the procedure. 8% also stops aspirin. 13% do not stop either of these medications before the procedure. (Fig. 4)

### Consent before the procedure

Only 52% of operators obtain written consent before TDC removal with the rest proceeding after verbal informed consent.

### Patient positioning during procedure

63% of operators prefer to have the patient with head above the level of the feet, 32% keep their patients lying flat, 4% report a position of head below the level of the feet.

### Sterility

69% of operators report performing a fully sterile procedure including sterile gloves, gown and drapes. The rest of the operators describe a clean but not fully sterile technique with any combination of non-sterile gloves, drapes, gown or apron.

### Procedure assistants

78% have a non-doctor assistant helping. 16% report they are left alone with the patient. 6% usually have another doctor assisting.

### Major complications experienced

21 out of 71 operating survey participants (30%) report experiencing a stuck catheter at some point which was removed either by the surgical or interventional radiology (IR) teams. 1 participant described “failure to remove requiring surgical assistant”. 4 reported other complications including; one retained cuff with traction method, one haematoma with a patient who was on warfarin, one cut line and one arterial bleeding. 13 participants do not know what local facilities they have available to them in the event of a stuck catheter but 22 report IR, 4 report surgical availability and 36 report both. No participant reported experiencing air embolism as a complication.

### Training curriculum

51 survey participants (68%) believe this procedure should be part of mandatory renal training curriculum and should be taught formally to be signed off. 20 participants (27%) are happy with the informal training they received. 4 participants gave other mixed responses.

## Discussion

Our survey suggests tunnelled haemodialysis catheter removals are predominantly performed by nephrology physicians in renal units in the UK. It is a common procedure with some nephrology registrars removing more than 10 catheters a year. TDC removals are generally safe with low complications rates as discussed above. However, there are some rare but significant complications to be aware of, namely stuck catheter, migration into intravascular system and air embolism. 30% of registrars who perform this procedure report experiencing stuck catheters at some point. Considering the major complications that may arise, the authors recommend obtaining written consent to show informed consent was obtained for medicolegal purposes.

There is little or none freely available written guidance from national or international bodies and a majority of local bodies. The majority of registrars were taught by other registrars.

Our survey highlights that there are practice differences across the UK in management of aspirin, antiplatelets and anticoagulation before TDC removal. Our survey also reports practice differences in patient positioning and the degree of sterility during the procedure.

There is not much published data on management of the antiplatelets and anticoagulants around the procedure. Dossabhoy et al. [8] report no increased risk of bleeding with patients who are on aspirin, clopidogrel or warfarin. However, the number of patients who had TDC removal on clopidogrel or warfarin were small. Furthermore, one of the three patients on warfarin received fresh frozen plasma before the procedure. Another study [19] investigated coagulation status and the time to achieve haemostasis after traction removal of TDC in 179 patients. They concluded that routine blood test for coagulation profile before the procedure was not necessary. They suggested that the antiplatelet use prolonged time to reach haemostasis. In practice, this only means applying pressure at previous venotomy site for longer. Interestingly there was no statistically significant increase in time to haemostasis with warfarin but this group was smaller than the antiplatelet group. There were ten patients whose INR was more than 2 and they all achieved haemostasis within the 5-minute cut off frame. There is clearly a need to investigate this area further as unnecessary cessation of clinically indicated anticoagulation may lead to harm.

Regarding patient positioning, whilst Trendelenburg position is widely recommended in temporary non-tunnelled catheter removal to prevent air embolism, this may not be strictly needed for tunnelled catheter removal as discussed above. The patient positioning with the head above the level of the feet can help keep the patient more comfortable.

The need for fully sterile personal protective equipment during the procedure should also be considered. Our survey found that 31% of operators do not follow full sterility during TDC removal. Carrying out the procedure with a non-sterile PPE but clean method may help reduce costs and wastage. More data is needed to establish if fully sterile PPE during the procedure reduces infection rates.

Finally, one of the most important findings was that 68% of participants think this procedure should be part of mandatory renal training curriculum and formally taught in UK postgraduate renal speciality training. Currently the training is provided informally and trainees are learning from peers with similar levels of clinical experience.

There are limitations to this survey. We received only 75 responses despite months of proactive advertisement through a variety of channels. We requested information under Freedom of Information Act 2000 from GMC. As a result, we are aware that there were 442 nephrology trainees registered at the time across the UK. Unfortunately, we were not able to deduce how many registrars were in active training as opposed to being out-of-programme due to a variety of reasons including long term sickness, maternity/paternity leave or other reasons. 21% of responses are from East Midlands representing the training region of the primary author. One likely explanation for a higher participation rate is that knowing the author in person may make it more likely for the participants to engage with the survey. As previously reported in a Canadian paper, there is survey fatigue among doctors with overall survey response rates of around 35% [20]. In UK, the General Medical Council (GMC) 2023 national training survey which is not mandatory but considered to be a professional obligation to complete, had a 74% completion rate in training doctors and 38% completion rate in trainers according to their own website.

Some survey participants work in the same hospitals together therefore this survey does not represent 75 individual renal units. The place of work was an optional question and the names of 29 different hospitals were given.

## Conclusions

In summary, tunnelled haemodialysis catheter removal is a routine procedure undertaken by renal physicians with different approaches across the UK. It must be performed in a safe way to minimise the risk of major complications. It is the authors opinion that the trainees should be supported to learn how to perform the procedure correctly as well as to learn how to deal with complications that may arise. There is a lack of formal training and guidance in this area across UK which needs to be addressed. Adding the procedure into the renal curriculum is advised as well as development of removal protocols including need for consent, consideration of medication cessation and descriptions of recommended methods at site of work.

## Electronic supplementary material

Below is the link to the electronic supplementary material.

Supplementary Material 1

## Data Availability

The datasets used and/or analysed during the current study are available from the corresponding author on reasonable request.

## Abbreviations

ACP: Advanced care practitioner
ASDIN: American Society of Diagnostic and Interventional Nephrology
CDM: Cut down method
DOAC: Direct oral anticoagulants
GAfREC: Governance arrangements for research ethics committees
GMC: General Medical Council
IR: Interventional Radiology
JRCPTB: The Joint Royal Colleges of Physicians’ Training Board (UK)
KDOQI: Kidney Disease Outcomes Quality Initiative
MCDM: Modified cut down method
PPE: Personal protective equipment
SHO: Senior house officer
SpR: Speciality registrar
ST: Speciality trainee
TDC: Tunnelled haemodialysis Catheter
TM: Traction method
TPD: Training programme directors
UK: United Kingdom
UKKA: UK Kidney Association

## References

[1] Letachowicz K, Gołębiowski T, Kusztal M, Penar J, Letachowicz W, Weyde W, et al. Over-catheter tract suture to prevent bleeding and air embolism after tunnelled catheter removal. J Vasc Access. 2017;18(2):170–2. 27834456 10.5301/jva.5000620

[2] Porazko T, Hobot J, Ziembik Z, Klinger M. Tunnelled haemodialysis catheter removal: an underappreciated problem, not always simple and safe. Int J Environ Res Public Health. 2020;17(9):3027. 32349262 10.3390/ijerph17093027 PMC7246895

[3] Kohli MD, Trerotola SO, Namyslowski J, Stecker MS, McLennan G, Patel NH, et al. Outcome of polyester cuff retention following traction removal of tunneled central venous catheters. Radiology. 2001;219(3):651–4. 11376249 10.1148/radiology.219.3.r01jn05651

[4] Fülöp T, Tapolyai M, Qureshi NA, Beemidi VR, Gharaibeh KA, Hamrahian SM, et al. The safety and efficacy of bedside removal of tunneled Hemodialysis catheters by nephrology trainees. Ren Fail. 2013;35(9):1264–8. 23924372 10.3109/0886022X.2013.823875

[5] Niyyar VD, Work J. Avoiding a Cutdown—Use of the transcatheter extractor in removal of tunneled Dialysis catheters. Semin Dial. 2011;24(1):115–7. 21324002 10.1111/j.1525-139X.2011.00847.x

[6] Fülöp T, Tapolyai MB, Agarwal M, Lopez-Ruiz A, Molnar MZ, Dossabhoy NR. Bedside tunneled Dialysis catheter Removal-A lesson learned from nephrology trainees. Artif Organs. 2017;41(9):810–7. 28025835 10.1111/aor.12869

[7] Fülöp T, Rodríguez B, Kosztaczky BA, Gharaibeh KA, Lengvárszky Z, Dossabhoy NR, et al. Tunneled Hemodialysis catheter removals by Non-Interventional nephrologists: the university of Mississippi experience. Semin Dial. 2015;28(5):E48–52. 25784000 10.1111/sdi.12364

[8] Dossabhoy NR, Sangha B, Tapolyai MB, Fülöp T. Outpatient removal of tunneled Dialysis catheters by nephrology fellows in training at a veterans affairs medical center. J Vasc Access. 2016;17(4):340–4. 27312761 10.5301/jva.5000571

[9] Vellanki VS, Watson D, Rajan DK, Bhola CB, Lok CE. The stuck catheter: A hazardous twist to the meaning of permanent catheters. J Vasc Access. 2015;16(4):289–93. 25953209 10.5301/jva.5000392

[10] Sequeira A, Sachdeva B, Abreo K. Uncommon complications of Long-Term Hemodialysis catheters: adhesion, migration, and perforation by the catheter tip. Semin Dial. 2010;23(1):100–4. 20331826 10.1111/j.1525-139X.2009.00681.x

[11] Hong JH. A breakthrough technique for the removal of a Hemodialysis catheter stuck in the central vein: endoluminal balloon dilatation of the stuck catheter. J Vasc Access. 2011;12(4):381–4. 21688240 10.5301/JVA.2011.8415

[12] Quaretti P, Galli F, Fiorina I, Moramarco LP, Spina M, Forneris G, et al. A refinement of Hong’s technique for the removal of stuck Dialysis catheters: an easy solution to a complex problem. J Vasc Access. 2014;15(3):183–8. 24190073 10.5301/jva.5000186

[13] Hong JH. An easy technique for the removal of a Hemodialysis catheter stuck in central veins. J Vasc Access. 2010;11(1):59–62. 20119909 10.1177/112972981001100112

[14] Forneris G, Savio D, Quaretti P, Fiorina I, Cecere P, Pozzato M, et al. Dealing with stuck Hemodialysis catheter: state of the Art and tips for the nephrologist. J Nephrol. 2014;27(6):619–25. 25319545 10.1007/s40620-014-0150-4

[15] Reddy A, Stangl A, Radbill B. Retained catheter fragment from a fractured tunneled catheter-A rare and potentially lethal complication. Semin Dial. 2010;23(5):536–9. 21039881 10.1111/j.1525-139X.2010.00756.x

[16] Kelly MJ, Anwar S, Vachharajani T, Karasek M, Ahmed S. LEARNING FROM IMAGES fundamental mistake during tunneled Hemodialysis catheter (TDC) removal. Open Urol Nephrol J. 2016;9(1):4–5.

[17] UKKA. Vascular Access for Haemodialysis 2023 [Available from: https://ukkidney.org/health-professionals/guidelines/guidelines-commentaries

[18] Lok CE, Huber TS, Lee T, Shenoy S, Yevzlin AS, Abreo K, et al. KDOQI clinical practice guideline for vascular access: 2019 update. Am J Kidney Dis. 2020;75(4):S1–164. 32778223 10.1053/j.ajkd.2019.12.001

[19] Stecker MS, Johnson MS, Ying J, McLennan G, Agarwal DM, Namyslowski J, et al. Time to hemostasis after traction removal of tunneled cuffed central venous catheters. J Vasc Interv Radiol. 2007;18(10):1232–9. 17911513 10.1016/j.jvir.2007.06.035

[20] Cunningham CT, Quan H, Hemmelgarn B, Noseworthy T, Beck CA, Dixon E et al. Exploring physician specialist response rates to web-based surveys. BMC Med Res Methodol. 2015;15(1). 10.1186/s12874-015-0016-z PMC4404667 25888346

