# Supplementary material for: Differences in training and practice in tunnelled haemodialysis catheter removal: a survey of nephrology trainees across United Kingdom
Source: BMC Nephrol. 2025 Mar 6;26:124. doi: 10.1186/s12882-025-04034-1 (PMC11887321; doi:10.1186/s12882-025-04034-1)
Supplement: Supplementary file 1 — Supplementary Material 1 [file 12882_2025_4034_MOESM1_ESM.pdf]

# Differences in practice and training in tunnelled dialysis catheter removal within Renal Registrars across UK

**This survey should take you 5 minutes to complete.**

The removal of tunnelled dialysis catheter is a necessary basic skill for the renal trainee as this task is undertaken routinely in renal units after successful establishment of other permanent access or due to need for removal of plastic in catheter-related infection. Anecdotally, most trainees suggest that they do not have sufficient supervised training in this clinical procedure, yet they are asked to take tunnelled lines out routinely.

There are different methods described in the literature. **We would like to establish the differences in practice and training across UK.** This survey is anonymous and we are not collecting any participant data beyond the information you are providing in your answers below. By completing this survey, you consent to participate in this study.

One method is referred to as the Cut-Down Method (CDM). In summary palpation is used to locate the cuff. A sterile field created around the cuff. Local anaesthetic is inserted to the skin above the cuff, then an 2-3cm incision to the skin is made over the cuff. The operator then blunt dissects down to expose the cuff using forceps and swabs until the sheath around the cuff is visible. Both top and bottom of the cuff are bluntly removed from the sheath round the catheter to fully mobilise the cuff before clamping either side. The catheter is then cut distal to the cuff and the central part removed from the vein. Pressure must be applied to the vein at this stage. The distal portion of the catheter can be removed from the exit site. 2 stitches will be needed to close the skin.

Another method is referred to as the Tractional Method (TM) where the operator applies the local anaesthetic in and around the exit site, and blunt dissects with instruments through the exit site to reach the cuff, all the while applying traction on the catheter with the other hand. The cuff is then liberated from the surrounding tissue with further blunt dissection and the whole catheter is pulled out with a controlled amount of vigorous force in one piece.

* Indicates mandatory question to complete

1. What is your training grade in Renal Medicine? If you are out-of-programme but have a training number please still select the grade that applies. *

*Mark only one.*

- ST3
- ST4
- ST5
- ST6
- ST7
- Not in training eg trust grade doctor, SAS, LAT
- Other: ____________

2. Which region are you currently working in? *

*Mark only one.*

- East Midlands
- West Midlands
- North East
- North West
- Yorkshire and Humber
- East of England
- South West
- Thames Valley
- Wessex
- London
- Kent, Surrey and Sussex
- Scotland
- Wales
- Northern Ireland
- Other: ____________

3. Which trust or hospital do you currently work for? (not a compulsory question)

________________________________________

4. Who removes the tunnelled dialysis catheters in your current Renal unit? (eg in case of line sepsis or upon maturation of AVF. This question is not related to line exchange) *
Tick ALL that applies

*Tick all that apply.*

- ☐ Renal Registrar
- ☐ Renal Consultant
- ☐ Nurse
- ☐ Interventional radiologist
- ☐ Other:

5. Where and in which deanery did you actually learn the tunnelled dialysis catheter removal? *

*Mark only one.*

- ◯ I was not taught at all and I do not perform this procedure.
- ◯ East Midlands
- ◯ West Midlands
- ◯ North East
- ◯ North West
- ◯ Yorkshire and Humber
- ◯ East of England
- ◯ South West
- ◯ Thames Valley
- ◯ Wessex
- ◯ London
- ◯ KSS
- ◯ Scotland
- ◯ Wales
- ◯ Northern Ireland
- ◯ Outside UK
- ◯ Other: ____________________________

6. Who taught you how to remove tunnelled dialysis catheters? *

*Mark only one.*

- ◯ Another renal registrar
- ◯ Renal Consultant
- ◯ Nurse
- ◯ Other: ______________________

7. How many tunnelled catheter removals have you performed so far? *

*Mark only one.*

- ◯ 0
- ◯ 1-9
- ◯ 10-50
- ◯ >50

8. How often are you asked to remove a tunnelled dialysis catheter a year? *

*Mark only one.*

- ◯ 0
- ◯ 1-5
- ◯ 6-10
- ◯ >10

9. There may be differences in the nature of the lines used, necessitating different ways of removal. What type of tunnelled dialysis catheters do you use? Tick all that applies (not a compulsory question)

*Tick all that apply.*

- ☐ Palindrome
- ☐ Tesio
- ☐ Other: ______________________

10. Which method do you practice? *

*Mark only one.*

- ( ) Cut-down method (mostly) ie a superficial small incision near the cuff site followed by the blunt dissection until the cuff is exposed.
- ( ) Tractional method (mostly) ie blunt dissection through the exit site with blunt instrument, releasing soft tissue around the catheter and the cuff while applying traction onto the catheter
- ( ) I can perform either and pick a method depending on the cuff position/patient factors
- ( ) Other: ______________________

11. Do you know if your trust has written guidance on tunnelled catheter removal? *

*Mark only one.*

- ( ) Yes, I read the tunnelled catheter removal guideline
- ( ) I never saw the relevant guideline but I know it exists
- ( ) I am not sure if we have the guideline, I never had to check.
- ( ) We do not have a guideline on tunnelled catheter removal

12. Where do you carry out the procedure? Multiple answers are allowed *

*Tick all that apply.*

- [ ] By the bedside on the ward/ or by the dialysis chair on the unit
- [ ] In a designated procedure area on the ward or on the dialysis unit
- [ ] Other: ______________________

13. Do you ask for these medications to be stopped in an elective removal? We accept that when stopped they are stopped for at least 5 days before the procedure. *

*Tick all that apply.*

- [ ] Don't stop either
- [ ] Aspirin
- [ ] Antiplatelets (clopidogrel, ticagrelor, prasugrel)
- [ ] Warfarin
- [ ] DOAC

14. Consent from the patient *

*Mark only one.*

- ( ) Written consent is obtained
- ( ) Verbal consent only.

15. How do you aim to position your patient in JUGULAR catheter removal? A suggestion is that whilst Trendelenburg position is important during catheter insertion to avoid air embolus, this may not be as important in removal and positioning at 45 degrees can reduce bleeding*

*Mark only one.*

- ( ) Head above the level of feet
- ( ) Head at the level of feet
- ( ) Head below the level of feet
- ( ) Other: ____________________

16. During the procedure, do you wear full PPE with a sterile gown and sterile gloves OR do you follow a clean but not sterile technique with an apron with nonsterile gloves? If anything in between, please describe*

*Mark only one.*

- ( ) Fully sterile with sterile gloves, gown, sterile drapes etc
- ( ) Clean technique but not sterile eg nonsterile apron/gloves, no drapes
- ( ) Other: ____________________

17. Do you have an assistant during the procedure or are you left completely alone with the patient? *

*Mark only one.*

- ( ) I usually have at least one non-doctor assistant
- ( ) I usually have another doctor assisting or supervising
- ( ) I am usually left alone with the patient

18. Have you had any major complications eg all or parts of the catheter retained in the patient's body, cuff retained under the skin, wound infection, stuck catheter, air embolus etc If yes, please describe. (not a compulsory question)

______________________________

19. In the event of a "Stuck catheter" ie the catheter is adhered to the vessel wall or right atrial wall with fibrin sheath, what support do you have locally? *

*Tick all that apply.*

- [ ] Surgical team for open surgery
- [ ] Interventional radiology team
- [ ] I dont know/ I am unsure
- [ ] Other: ______________________________

20. Do you think this procedure should be a part of the renal training curriculum as a mandatory procedure so that the hospitals will have a further incentive to ensure this procedure is taught in a supervised manner assisting a standard of training across all trainees. *

*Mark only one.*

- ( ) Yes, I would prefer if this procedure was taught more formally.
- ( ) No, I am happy with informal training.
- ( ) Other: ______________________________
